# Temporal trends of main reproductive characteristics in ten urban and rural regions of China: the China Kadoorie Biobank study of 300 000 women

**DOI:** 10.1093/ije/dyu035

**Published:** 2014-03-17

**Authors:** Sarah Lewington, LiMing Li, Serini Murugasen, Lai-san Hong, Ling Yang, Yu Guo, Zheng Bian, Rory Collins, Junshi Chen, Hui He, Ming Wu, Tianyou He, Xiaolan Ren, Jinhuai Meng, Richard Peto, Zhengming Chen

**Affiliations:** ^1^Clinical Trial Service Unit and Epidemiological Studies Unit, University of Oxford, Oxford, UK, ^2^School of Public Health, Peking University Health Science Center, Beijing, China, ^3^Chinese Academy of Medical Sciences, Dong Cheng District, Beijing, China, ^4^Chinese National Center for Food Safety Risk Assessment, Beijing, China, ^5^Nangang Center for Disease Control, Haerbin, Heilongjiang, China, ^6^Jiangsu Centre for Disease Control, Nanjing, Jiangsu, China, ^7^Huixian Centre for Disease Control, Huixian, Hennan, China, ^8^Gansu Centre for Disease Control, Lanzhou, Gansu, China and ^9^Liuzhou Centre for Disease Control, Liuzhou, Guangxi, China

**Keywords:** Reproductive, temporal trends, China, cohort study

## Abstract

**Background:** Chinese women’s reproductive patterns have changed significantly over the past several decades. However, relatively little is known about the pace and characteristics of these changes either overall or by region and socioeconomic status.

**Methods:** We examined the cross-sectional data from the China Kadoorie Biobank cohort study that recruited 300 000 women born between 1930 and 1974 (mean age: 51 years) from 10 socially diverse urban and rural regions of China. Temporal trends in several self-reported reproductive characteristics, and effect modification of these trends by area and education (as a surrogate for socioeconomic status), were examined.

**Results:** The overall mean age at menarche was 15.4 (standard deviation 1.9) years, but decreased steadily over the 45 birth cohorts from 16.1 to 14.3 years, except for an anomalous increase of ∼1 year for women exposed to the 1958-61 famine in early adolescence. Similarly large changes were seen for other characteristics: mean parity fell (urban: 4.9 to 1.1; rural: 5.9 to 1.4); mean age at first birth increased (urban: 19.0 to 25.9 years; rural: 18.3 to 23.8 years); and birth spacing increased after 1980 to over 5 years. Breastfeeding declined after 1950 in urban and, after 1980, in rural women; and 68% of urban and 48% of rural women experienced a terminated pregnancy. Mean age at menopause increased from 47.9 to 49.3 years.

**Conclusions** There have been striking changes in reproductive factors over time and between areas among these Chinese women. Their effects on major chronic diseases should be investigated.

**Key Messages**

- The reproductive patterns of Chinese women have changed significantly over the past several decades, partly or wholly due to the major social and economic changes that have occurred in China.
- Mean age at menarche decreased by approximately 2 years between women born in 1930 and those born in 1974.
- Women who experienced the 1958-61 famine around puberty had, on average, a 1-year delay in menarche, with somewhat greater effect among the rural and the least educated women. In contrast, there was no interruption in the downward trend for women who experienced famine around the time of birth (either *in utero* or in the first few months of life).
- Parity fell steadily from 1950 (as in other surrounding countries), with no obvious inflection after the introduction of the one-child policy, suggesting that fertility rates may have eventually fallen to below replacement level without strict fertility control, particularly in some urban areas.
- The main effect of the one-child policy seems to have been to increase the mean age at first birth, especially in urban areas, and there was a sudden increase in the spacing between first and second births after its introduction.

## Introduction

The reproductive patterns of Chinese women have changed significantly over the past several decades, partly or wholly due to the major social and economic changes that have occurred in China.^1^^,^^2^ These changes include: the introduction of a strict family planning policy (the so-called ‘one-child’ policy); recent rapid urbanization and economic development; and periodic social upheavals (e.g. wars, famines). However, the pace and characteristics of these changes—either overall or by region and socioeconomic status—have not been properly investigated, nor have the potential long-term consequences of these changes on major chronic diseases.

In China, a strict family planning policy was introduced during the late 1970s, which not only restricts the number of children a couple can have (usually one in urban and up to two in rural areas) but also encourages late marriage and childbirth. The seemingly large and profound impact on reproductive patterns of Chinese women has not always been considered in the context of other rapid social and demographic changes seen since the 1950s.^1^

Studies from predominantly Western populations have shown that reproductive characteristics change as countries become more affluent and better educated.^3^ As a result of improved nutrition, the mean age at menarche falls and at menopause rises,^4^ and as women become more educated, the fertility rate declines^5^ as well as the likelihood and duration of breast feeding.^6^

There is evidence from the West that exposure to famine can cause a sharp, but temporary, increase in mean age at menarche,^7^ modestly decreased reproductive function^8^ and a small decrease in age at natural menopause.^9^ However, there are only limited Chinese data on the effects of famine on reproductive factors.^10^

Although there have been reports of temporal changes in reproductive characteristics among Chinese women,^1^^,^^2^ they have been small, from limited geographical areas or were conducted many years ago, and have been unable to assess reliably whether the pace of change was affected by urbanization and socioeconomic status. We report data on 300 000 women from the China Kadoorie Biobank study (CKB),^11^ who were recruited during 2004-08 from 10 socially diverse areas of China. These women were born between 1925 and 1978 and so experienced the major social and economic changes described during their reproductive lives (1950s to 1990s). We aim to examine the temporal trend of reproductive characteristics both overall and by urbanization, and to assess the impact of major socioeconomical factors on any such trends.

## Methods

The CKB study is a blood-based prospective cohort study of 0.5 million Chinese adults. Detailed information about the study design and procedures has been reported previously.^11^^,^^12^ Briefly, the baseline survey took place from 2004 to 2008 in 10 geographically defined areas of China (eFigure 1, available as Supplementary data at *IJE* online). At the baseline survey, socio-demographic, lifestyle and medical data were collected. Women were asked about their reproductive history, including age at menarche and menopause, gravidity and parity, duration of breastfeeding for each live birth, use of oral contraceptive pills and history of hysterectomy, ovarian or breast surgery. All participants are being followed up for cause-specific morbidity and mortality but only the baseline data have been used for this report to perform a retrospective analysis of the women’s reproductive histories.

Ethical approval was obtained from the ethical review committee of the Chinese Center for Disease Control and Prevention, Beijing, China and the Oxford Tropical Research Ethics Committee, University of Oxford, UK. All study participants provided written informed consent.

### Statistical methods

Only women with known age at birth, known educational history and the relevant reproductive history data were included in these analyses. Analyses of childbearing characteristics were further restricted to women aged 35 years or over, of whom more than 95% had finished child bearing. Only women with at least one live birth were included in analyses of age at first birth and breastfeeding, and only women with at least two live births were included in analyses of time between first and second births. Analyses of menopause included only women aged 57 years or over (of whom 99% reported being post-menopausal) and who had experienced natural menopause. For analyses of breastfeeding, the average duration of breastfeeding per baby was calculated for each woman. For analyses of abortion (termination of pregnancy or miscarriage), the rate is the percentage of women reporting at least one abortion. The abortion rates differed greatly in Gansu (a rural area in central China: eFigure 1, available as Supplementary data at *IJE* online) from the other regions (see Results), and so have been presented separately.

To describe the population, crude distributions of age, education and the reproductive factors were calculated separately for women in urban and rural areas, and overall. To assess temporal trends, area-adjusted means of each reproductive factor were calculated by year-of-birth of the woman or by year-of-birth of the first child, whichever seemed most appropriate, and plotted as a 3-year moving average. Years with fewer than 100 women were not plotted because the results were too unreliable.

Women with inconsistent data on any reproductive factor (*n* = 166) were removed from the dataset and, to avoid extreme values (which may or may not have been data entry errors) skewing the results, women in the top and bottom 0.1% of values for any reproductive variable within a 5-year age band were also excluded (*n* = 1917). No attempt was made to impute values for this 0.7% of excluded data. All analyses were performed using SAS version 9.2.

## Results

Of 302 669 women recruited into the study, 300 586 had all the data required to include them in the analyses, and the main demographic and reproductive characteristics of these women are given in Table 1 (and, for each of the 10 areas, in eTable 1 available as Supplementary data at *IJE* online). The mean [standard deviation (SD)] baseline age was 51 (11) years and the mean year of birth was 1955. Urban and younger women tended to be better educated: one-third of all urban women completed high school, whereas only 6% of all rural women did so (one-third had no formal education at all). Over 99% of the women had ever been married, with 9% widowed and 2% divorced at the time of the baseline survey. 88% of the women reported experiencing menarche between ages 13 to 18, with mean (SD) of 15.4 (1.9) years. Almost all the women (99%) had experienced at least one pregnancy and >99% of those had at least one live birth. Urban women had fewer children on average and the mean age at first birth was older than for rural women (Table 1; both *P* <0.0001). Most women had breastfed at least one of their babies (97%), with 93% of them breastfeeding each baby for more than 6 months, on average. Among women who had breastfed, rural women breastfed for an average 4 months longer than urban women (16.4 vs 12.2 months, *P* <0.0001). Excluding Gansu, 58% of women had experienced terminated pregnancy (68% urban, 48% rural), whereas 8% of women had experienced a miscarriage (6% urban, 10% rural). The pattern in Gansu had very different rates: only 3% of women had experienced termination of pregnancy whereas 16% had miscarried. The mean (SD) age at menopause was 48.2 (4.3) years among the 83 791 women who were naturally post-menopausal at the time of interview, and was slightly higher in urban than rural women (48.5 vs 48.0 years, *P* <0.0001). These crude analyses, however, disguise temporal trends in all reproductive factors.

**Table 1.** Demographic and reproductive characteristics

Characteristics	Urban	Rural	Overall
**Number of women**	134 173	166 413	300 586
**Age (years)**			
Mean (SD)	52.1 (10.7)	50.0 (10.2)	50.9 (10.5)
**Birth cohort (%)**			
<1930	<1	<1	<1
1930–1939	13	8	10
1940–1949	21	20	21
1950–1959	32	32	32
1960–1969	28	34	31
>= 1970	5	6	6
Mean (SD)	1954 (10.7)	1956 (10.2)	1955 (10.5)
**Highest education (%)**			
No formal school	17	32	25
Primary school	20	40	31
Middle school	30	22	25
High school	23	6	14
University/College	9	<1	4
**Age at menarche (%)**			
<13	6	5	5
13–14	30	26	28
15–16	36	38	37
17–18	22	25	23
> = 19	5	6	6
Mean (SD)	15.3 (2.0)	15.5 (1.9)	15.4 (1.9)
**Number with at least one pregnancy**	132 456	165 333	297 789
**(excluding Gansu)**	–	135 188	267 644
**Number of live births (%)**			
0	<1	<1	<1
1	52	21	35
2	24	39	32
3–4	20	31	26
> = 5	4	9	7
Mean (SD)	1.9 (1.2)	2.5 (1.4)	2.2 (1.3)
Median (Q1, Q3)	1 (1, 2)	2 (2, 3)	2 (1, 3)
**History of abortion****^a^**			
No history of abortion	29	45	37
History of induced abortion^b^	68	48	58
History of spontaneous abortion^b^	6	10	8
**Number with at least one live birth**	131 613	165 006	296 619
**Age at first birth (%)**			
<20	4	13	9
20–21	11	27	20
22–23	19	29	24
24–25	28	21	24
> = 26	37	10	22
Mean (SD)	24.7 (3.3)	22.3 (2.7)	23.4 (3.2)
**Average duration of breastfeeding (%)**			
Never breastfed	5	1	3
1 – 6 months	9	4	7
7 – 12 months	59	37	47
13 – 18 months	17	26	22
19 – 24 months	7	21	15
> 24 months	3	10	7
Mean (SD)	12.2 (6.4)	16.4 (7.9)	14.5 (7.6)
Median (Q1, Q3)	12 (10, 13)	15 (12, 21)	12 (11, 18)
**Age at menopause (years)****^c^**			
Number of women	41 190	42 601	83 791
<43	8	11	9
43–47	22	24	23
48–53	58	54	56
>= 54	12	11	12
Mean (SD)	48.5 (4.3)	48.0 (4.4)	48.2 (4.3)

^a^Excludes Gansu because of very different rates (81% no abortion; 3% termination of pregnancy; 16% miscarriage).

^b^Some women will have experienced both termination of pregnancy and miscarriage.

^c^Excludes women <57 years old or who had surgical menopause (hysterectomy, oophorectomy) or who had a daiagnosis of cancer.

**Figure 1.**
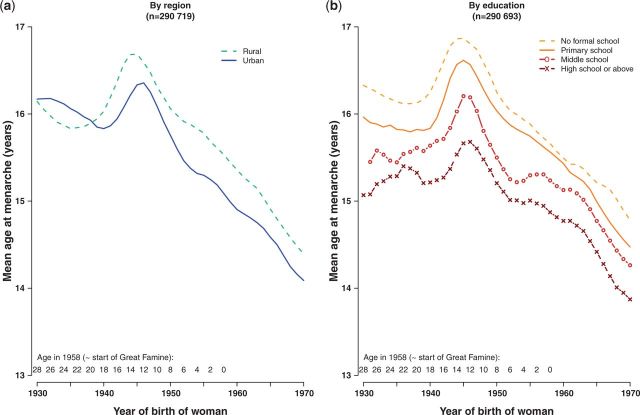
Time trends in mean age at menarche (a) by region and (b) by highest level of education

Figure 1 shows the temporal trend for mean age at menarche for women born 1930–70, by area and by highest level of education. Overall, mean age at menarche decreased from 16.1 to 14.3 years over 40 years. Although the downward trend was predominantly linear, there was an anomalous increase among women born around 1940–50, who were aged 8–18 years at the approximate start of the famines in 1958–61. The deviation in the temporal trend began earlier and lasted for longer in rural compared with urban women (Figure 1a). Education was a strong determinant of age at menarche, with the mean age at menarche decreasing with increasing level of education in every birth cohort (Figure 1b). Moreover, among the more educated women the anomalous increase due to famine was later and less extreme. This effect of education was more marked among urban women than among rural women (eFigure 2, available as Supplementary data at *IJE* online).

**Figure 2.**
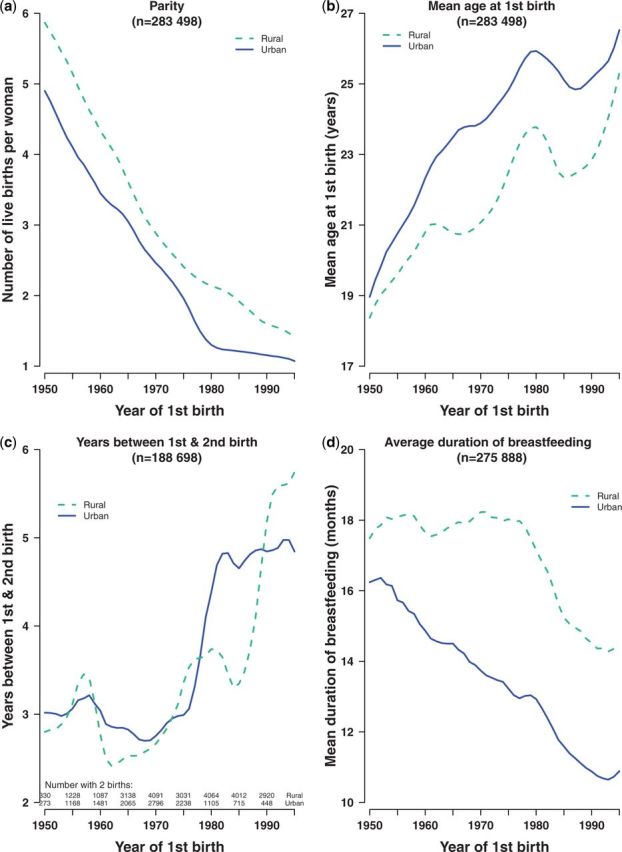
Time trends in reproductive characteristics, by region. (a) Parity, (b) mean age at first birth, (c) years between first and second birth and (d) mean duration of breastfeeding [All among women aged 35 years or over. (a) and (b): among women with at least one live birth; (c) among women with at least two live births; (d) among women who had ever breastfed]

Temporal trends in characteristics associated with childbearing are shown in Figure 2. In both urban and rural women, parity fell dramatically from 1950 onwards. These decreases were continuous with no sudden falls or plateaus until, among urban women, parity reached almost one (Figure 2a). In urban areas, higher education was associated with lower parity: in 1950, urban women with no formal education had two more children, on average, than women educated to high school or above; but this effect of education was greatly reduced by 1980 (eFigure 3, available as Supplementary data at *IJE* online). By contrast, there was no difference by education among rural women. Mean age at first birth increased over time, at least up to 1980, after which the mean age at first birth decreased again (Figure 2b). For urban women, this increase was steady and continuous over the 30-year period (from 19.0 to 25.9 years), but there was some fluctuation among rural women, with a small fall for first births in 1962–66, just after the end of the famine period. From 1980-85, there was a significant drop of about 1 year in the mean age at first birth among both urban and rural women, before it started to rise again. The peak occurred earlier and the fall was less extreme among the most highly educated urban women, whereas in rural women, education had less effect on the trend (eFigure 4, available as Supplementary data at *IJE* online).

Birth spacing showed a more unusual pattern (Figure 2c). Among urban mothers whose first birth was before 1975, there were just under 3 years, on average, between first and second babies, although there was a small temporary increase during the late 1950s. After 1975, the gap leapt rapidly to almost 5 years for women having their first child in 1982. This increase was greatest among the most highly educated urban women (to over 6 years between births: eFigure 5, available as Supplementary data at *IJE* online). The trend was more erratic among rural women: birth spacing eventually increased to a mean of 5.5 years regardless of education (eFigure 5, available as Supplementary data at *IJE* online).

Breastfeeding was more common and lasted longer in rural than in urban areas (Figure 2d). For urban women, the mean duration of breastfeeding fell steadily from 16 to 11 months. In rural areas, the mean lactation period was 18 months up to 1980, then dropped rapidly to 14 months. In both urban and rural areas, women who had completed high school were less likely to breastfeed than those with no formal schooling (94% vs 99%: data not shown) and, among those who did breastfeed, there was an inverse association with level of education, which diminished with time (eFigure 6, available as Supplementary data at *IJE* online).

**Figure 3.**
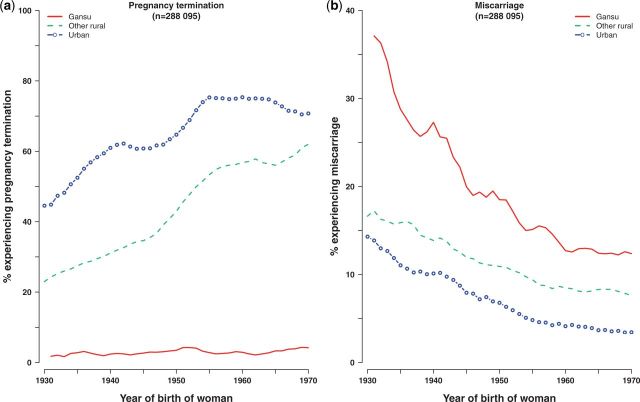
Time trends in termination of pregnancy and miscarriage, by region. Percentage experiencing event = number of women with at least one such event/total number of women

Figure 3 shows termination and miscarriage rates for all urban, Gansu and all other rural areas. Termination of pregnancy was far higher in urban than in rural areas for every year of birth, and the rate increased with year of birth from 45% to 71% among urban and from 23% to 62% among rural women (Figure 3a). By contrast, miscarriage fell from 14% to 3% among urban and 17% to 8% among rural women. In Gansu, termination rates were consistently very low (<5%) whereas miscarriage rates fell from 37% to 12%.

**Figure 4.**
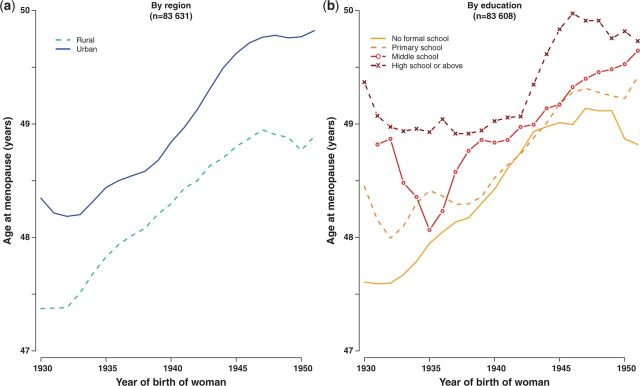
Time trends in mean age at menopause (a) by region and (b) by highest level of education

Age at menopause rose steadily in both urban and rural women, increasing by 11 [standard error (SE) 0.3] months for every 10-years-later year of the woman’s birth (i.e. from 47.9 to 49.3 years) but, across all birth cohorts, urban women experienced menopause on average 7 months later than rural women (Figure 4a). There was some association between education and age at menopause, with the most highly educated women experiencing menopause on average 1 year later than those with no education (Figure 4b).

## Discussion

In this study of 300 000 Chinese women born between 1930 and 1974 in 10 diverse regions of China, we observed some intriguing patterns over time and between social groups in all of the reproductive factors studied. On average, a woman in this study experienced menarche at 15 years of age, had two children (with the first live birth at 23 years) who were each breastfed for a mean of 14 months, and completed menopause at 48 years, giving her 33 years of reproductive life. However, this summary disguises significant differences between urban and rural women, by education and, more strikingly, changes over time.

Mean age at menarche decreased by approximately 2 years over the 45-year birth cohorts. This downward trend has been observed in many countries during the 20th century, but the pace and degree of the changes observed in these Chinese women are greater than in the UK or USA^13^ although comparable to Japan.^14^ Most striking, however, was the 1-year delay in menarche among the women who experienced famine around puberty. Although this peripubertal effect of famine on menarche has been observed in other studies,^7^^,^^14–16^ the women were drawn from small geographical areas and so it was not possible to investigate the effect of famine under different social and economic circumstances. In our study, the effect of famine on menarche was greater among the rural and the least educated women: the famine hit the urban areas later and was less severe, so it is perhaps not surprising that the delay in menarche occurred later and was less severe there. The dampening effect of education (as a proxy for social class) suggests that the more highly educated women were somewhat protected from the effects of famine. There was no interruption in the downward trend for women who experienced famine around the time of birth (either *in utero* or in the first few months of life). This is concordant with the Dutch famine birth cohort^17^ which also found no apparent association between perinatal famine exposure and age at menarche. It is not possible in a Chinese population to disentangle the long-term effects of pre-menarcheal famine (either around menarche or *in utero*) on reproductive function from the changes in other social conditions that occurred after the famine. Women who underwent puberty during the famine were having their first babies in the late 1960s, and there is no good evidence from these data to suggest that they suffered any long-term reproductive consequences of prepubescent famine. Women who were born during the famine were having their first babies during the 1980s, and so trying to disentangle any potential effects of *in utero* or neonatal famine from those of the fertility control measures imposed during the 1980s^1^ is virtually impossible.

Though not nationally representative, our findings suggest that the reproductive behaviour of these Chinese women was already changing well before the introduction of the one-child policy, probably due to China’s economic development and improved education.^12^ Parity (which is approximately equivalent to the fertility rate among these women, since 99% of them had at least one live birth) fell steadily from 1950, with no obvious inflection after the one-child policy was introduced, suggesting that fertility rates may have eventually fallen to below replacement level without strict fertility control, particularly in some urban areas. Indeed, during the latter half of the 20th century, fertility rates also fell in the neighbouring countries, so that by the end of the 20th century their total fertility rates were lower than in China: official data from the United Nations show that China’s total fertility rate was just over 1.8, compared with 1.1 in Hong Kong, 1.6 in Singapore and South Korea and 1.4 in Taiwan.^18^

As in high-income countries,^19^ these data show that mean age at first birth has increased significantly in China. The greater increase in mean age at first birth among urban women compared with rural women reflects the greater economic and social changes that have occurred as well as stricter implementation of the family planning policy in urban China. However, there was an anomalous fall in age for both urban and rural women having their first babies in the 1980s (that is, around the introduction of the one-child policy). This fall in mean age at first birth is intriguing and has not been reported elsewhere. Birth spacing shows the most striking pattern and is the one aspect of childbearing that appears to have been affected by both the famine and the fertility control policies. In both urban and rural women, there was a temporary rise and fall in the spacing between first and second births in the late 1950s, but more striking is the sudden increase among urban women at the end of the 1970s; the increase among rural women was less extreme and occurred about a decade later. This is in keeping with the greater enforcement of the family planning policies in urban areas.^20^ Finally, both the incidence and the duration of breastfeeding fell with time, in common with other studies observing this pattern as women become more economically active and better educated.^6^

In our study, the younger and more educated women experienced menopause later. There is no previous information from large-scale population-based studies in China, but limited data from developed countries suggest that the mean age at menopause seems to have either remained stable or increased slightly over the 20th century.^21^^,^^22^

The quality of the data collected in CKB is excellent, with very little missing information and good reproducibility.^23^ Moreover, any misreporting is likely to be random,^24^ which would attenuate any trends.^25^

The striking variations and rapid changes in Chinese women’s reproductive characteristics demonstrated by this large study may well have long-term consequences on major chronic diseases. Several smaller studies have shown associations between reproductive factors—such as age at menarche, age at first birth, breastfeeding and age at menopause—and risk of breast cancer.^14^^,^^26^^,^^27^ More controversially, gender differences in cardiovascular disease rates^2^ have prompted hypotheses that endogenous estrogen may play a role.^28^^,^^29^ However, few of these studies have been large enough on their own to quantify reliably the relevance of the major reproductive characteristics described here to risk, and there is extremely limited prospective evidence from China. With over 300 000 women and established linkages with mortality registries and coded hospitalization episodes,^12^ the CKB should soon be able to provide large-scale prospective evidence about the effects of reproductive characteristics on the risk of major chronic diseases in Chinese women.

## Supplementary Data

Supplementary data are available at *IJE* online.

## Funding

The baseline survey and the first re-survey were supported by a research grant from the Kadoorie Charitable Foundation in Hong Kong. The long-term continuation of the project during 2009–14 is supported by programme grants from the Wellcome Trust in the UK (088158/Z/09/Z] and the Chinese Ministry of Science and Technology (2011BAI09B01). The UK Medical Research Council, the British Heart Foundation (BHF) and Cancer Research UK also provide core funding to the Clinical Trial Service Unit and Epidemiological Studies Unit at Oxford University for the project.

## Supplementary Material

Supplementary Data
