# Supplementary material for: Temporal trends of main reproductive characteristics in ten urban and rural regions of China: the China Kadoorie Biobank study of 300 000 women
Source: Int J Epidemiol. 2014 Mar 17;43(4):1252–62. doi: 10.1093/ije/dyu035 (PMC4121552; doi:10.1093/ije/dyu035)
Supplement: Supplementary Data [file supp_43_4_1252__index.html]

Temporal trends of main reproductive characteristics in ten urban and rural regions of China: the China Kadoorie Biobank study of 300 000 women — Temporal trends of main reproductive characteristics in ten urban and rural regions of China: the China Kadoorie Biobank study of 300 000 women — Supplementary Data 

# Temporal trends of main reproductive characteristics in ten urban and rural regions of China: the China Kadoorie Biobank study of 300 000 women

## Supplementary Data

files

**Files in this Data Supplement:**

- Supplementary Data - pdf file
