# Supplementary material for: Temporal trends of main reproductive characteristics in ten urban and rural regions of China: the China Kadoorie Biobank study of 300 000 women
Source: Int J Epidemiol. 2014 Mar 17;43(4):1252–62. doi: 10.1093/ije/dyu035 (PMC4121552; doi:10.1093/ije/dyu035)
Supplement: Supplementary Data [file supp_dyu035_ije-2013-06-0615-File007.pdf]

# Patterns and temporal trends of main reproductive characteristics in urban and rural populations of China: China Kadoorie Biobank study of 300,000 women

## Supplementary material

**Supplementary figure 1. Locations of the China Kadoorie Biobank recruitment centres**

**Supplementary figure 2. Time trends in mean age at menarche, by education separately in (a) urban; (b) rural areas**

**Supplementary figure 3. Time trends in number of live births, by education separately in (a) urban; (b) rural areas**

Among women with at least one live birth.

**Supplementary figure 4. Time trends in mean age at first birth, by education separately in (a) urban; (b) rural areas**

Among women with at least one live birth.

**Supplementary figure 5. Time trends in years between first and second birth, by education separately in (a) urban; (b) rural areas**

Among women with at least two live births.

**Supplementary figure 6. Time trends in mean duration of breast-feeding, by education separately in (a) urban; (b) rural areas**

Among women who had ever breastfed.

**Supplementary figure 7. Time trends in induced abortion, by education separately in (a) urban; (b) rural areas**

% experiencing abortion = number of women with at least one abortion/total number of women

**Supplementary figure 8. Time trends in spontaneous abortion, by education**

% experiencing abortion = number of women with at least one abortion/total number of women

**Supplementary table 1. Demographic and reproductive characteristics in each of the 10 areas**

**Supplementary figure 1: Locations of the China Kadoorie Biobank recruitment centres**

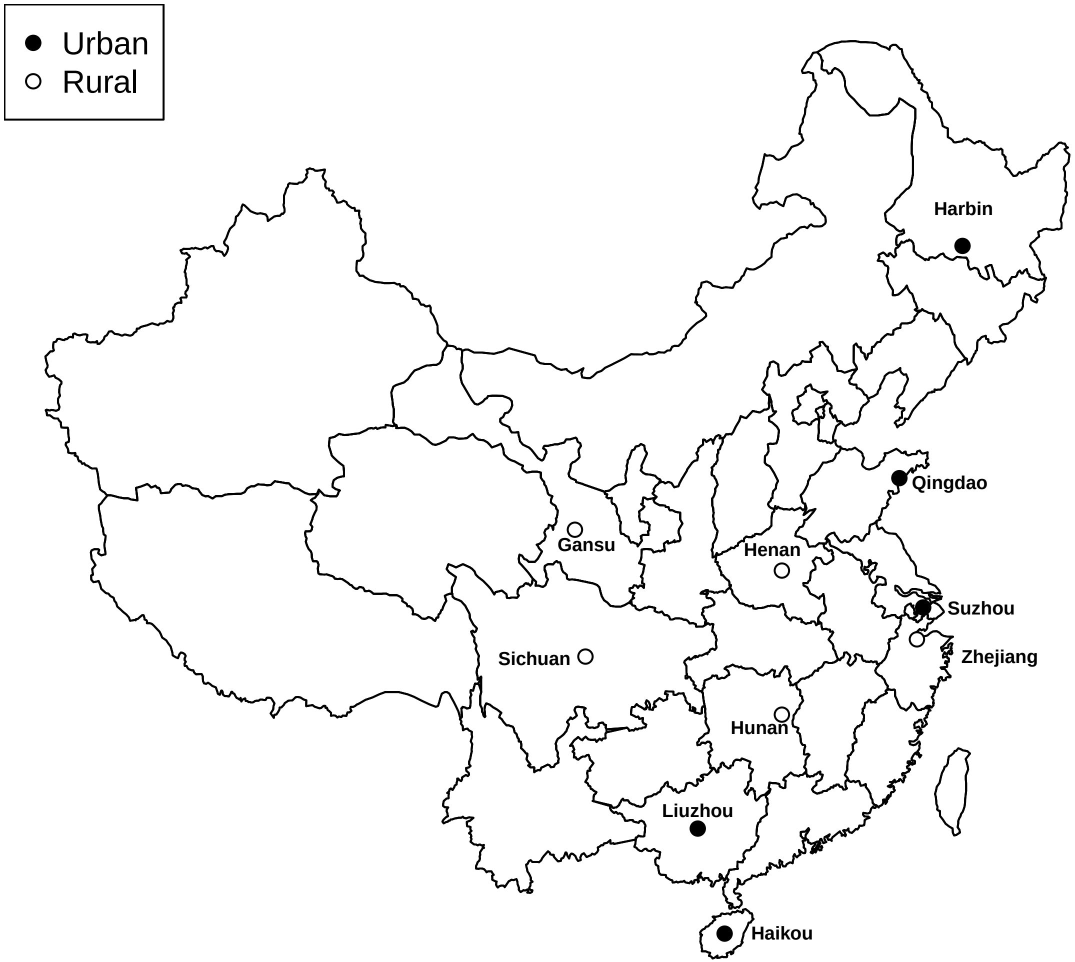

**Supplementary Figure 2. Time trends in mean age at menarche in urban & rural regions, by education**

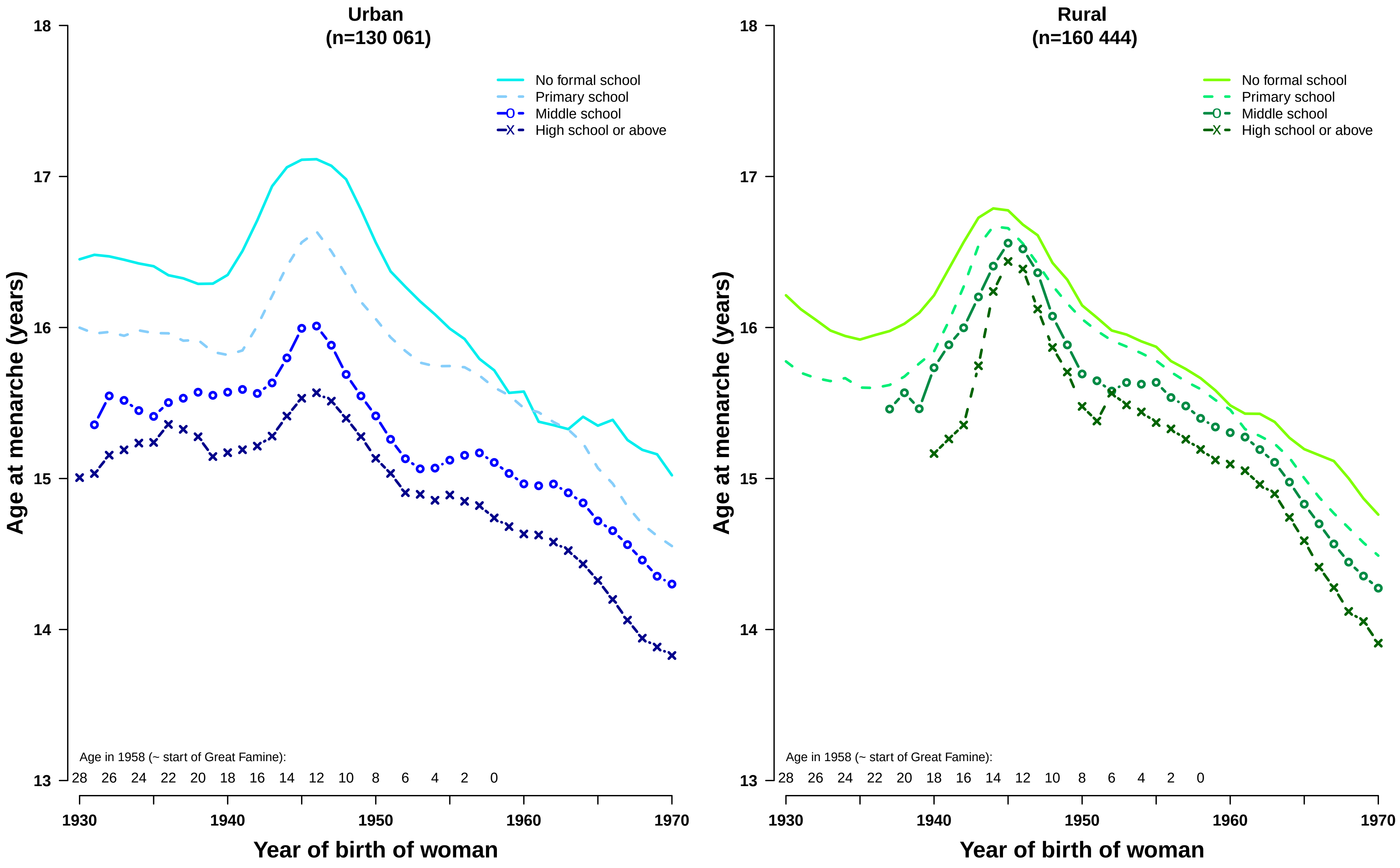

**Supplementary Figure 3. Time trends in number of live births in urban & rural, by eduation**

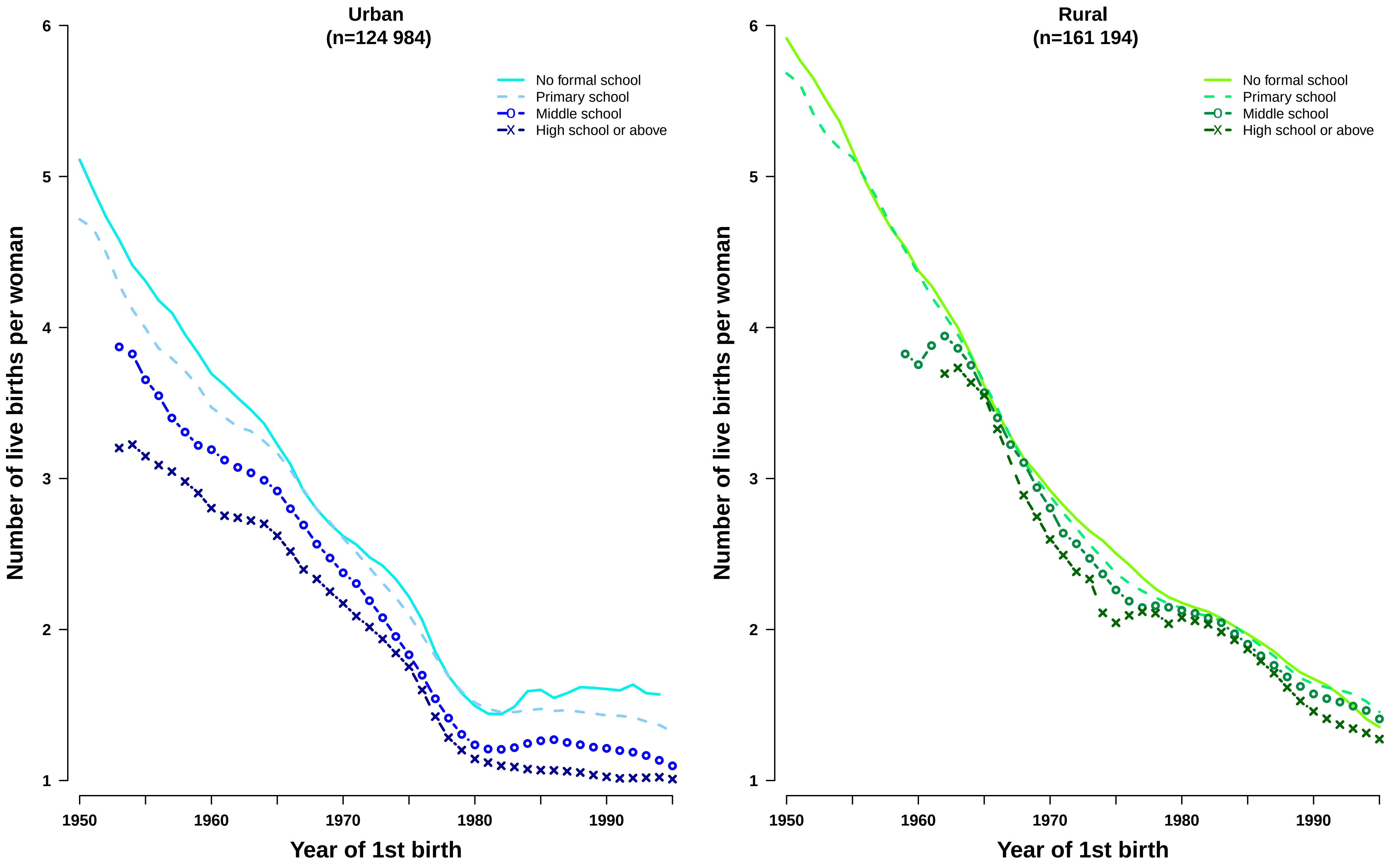

Supplementary Figure 4. Time trends in mean age at 1st birth in urban & rural, by education

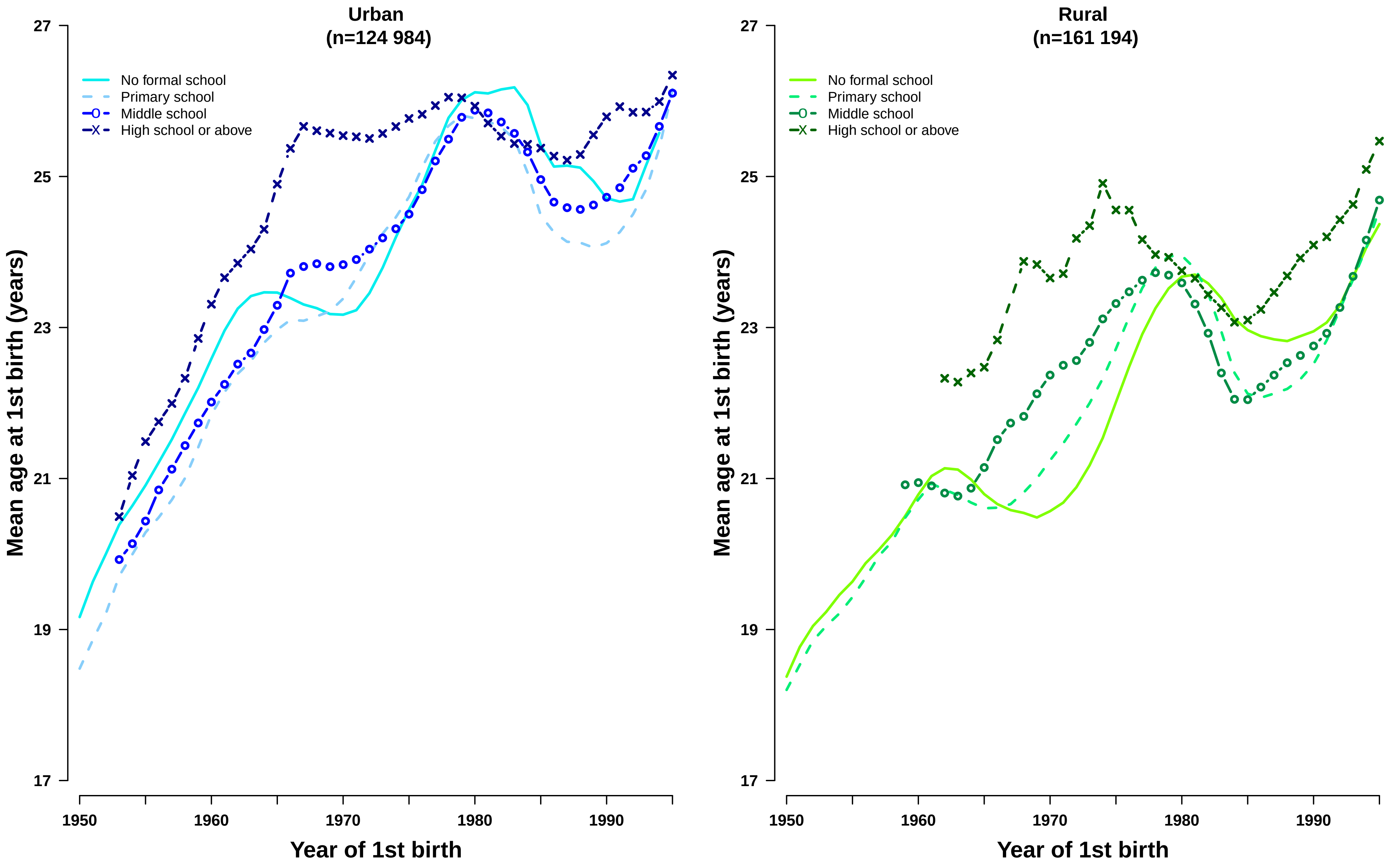

**Supplementary Figure 5. Time trends in years between 1st & 2nd birth in urban & rural, by education**

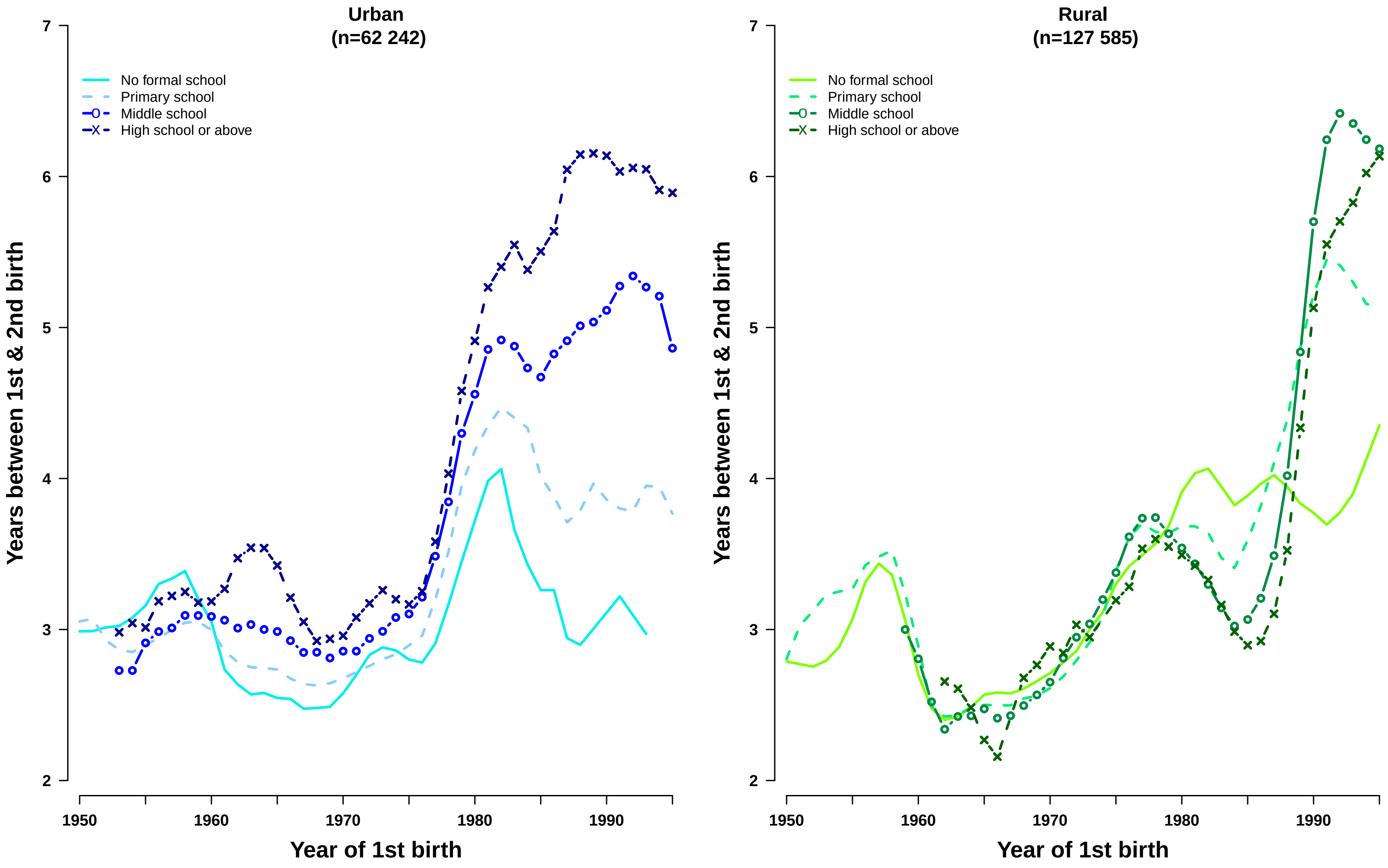

**Supplementary Figure 6. Average duration of breast−feeding by year of 1st birth**

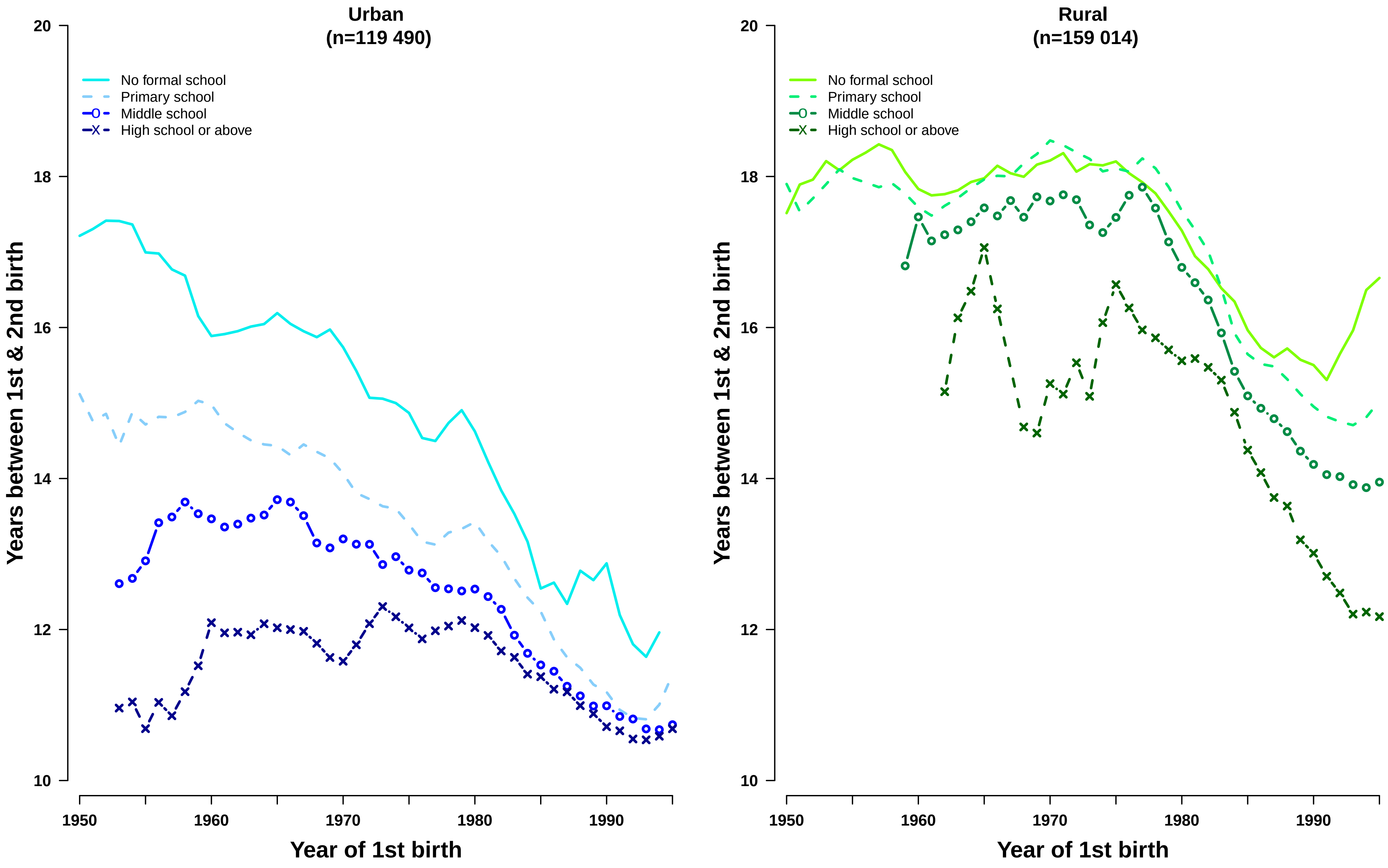

## Supplementary Figure 7. Time trends in induced abortion in different regions, by education

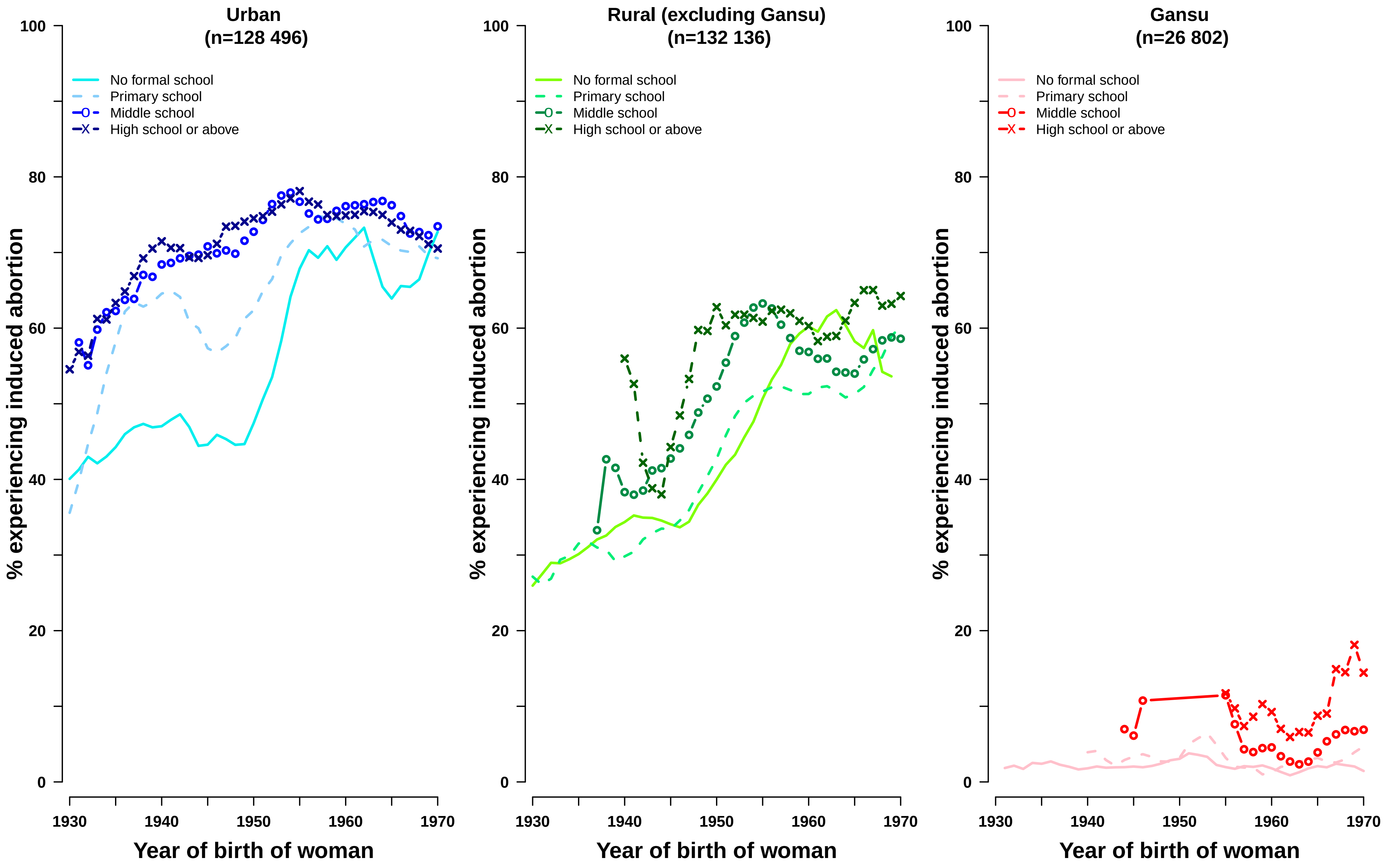

**Supplementary Figure 8. Time trends in spontaneous abortionin different regions, by education**

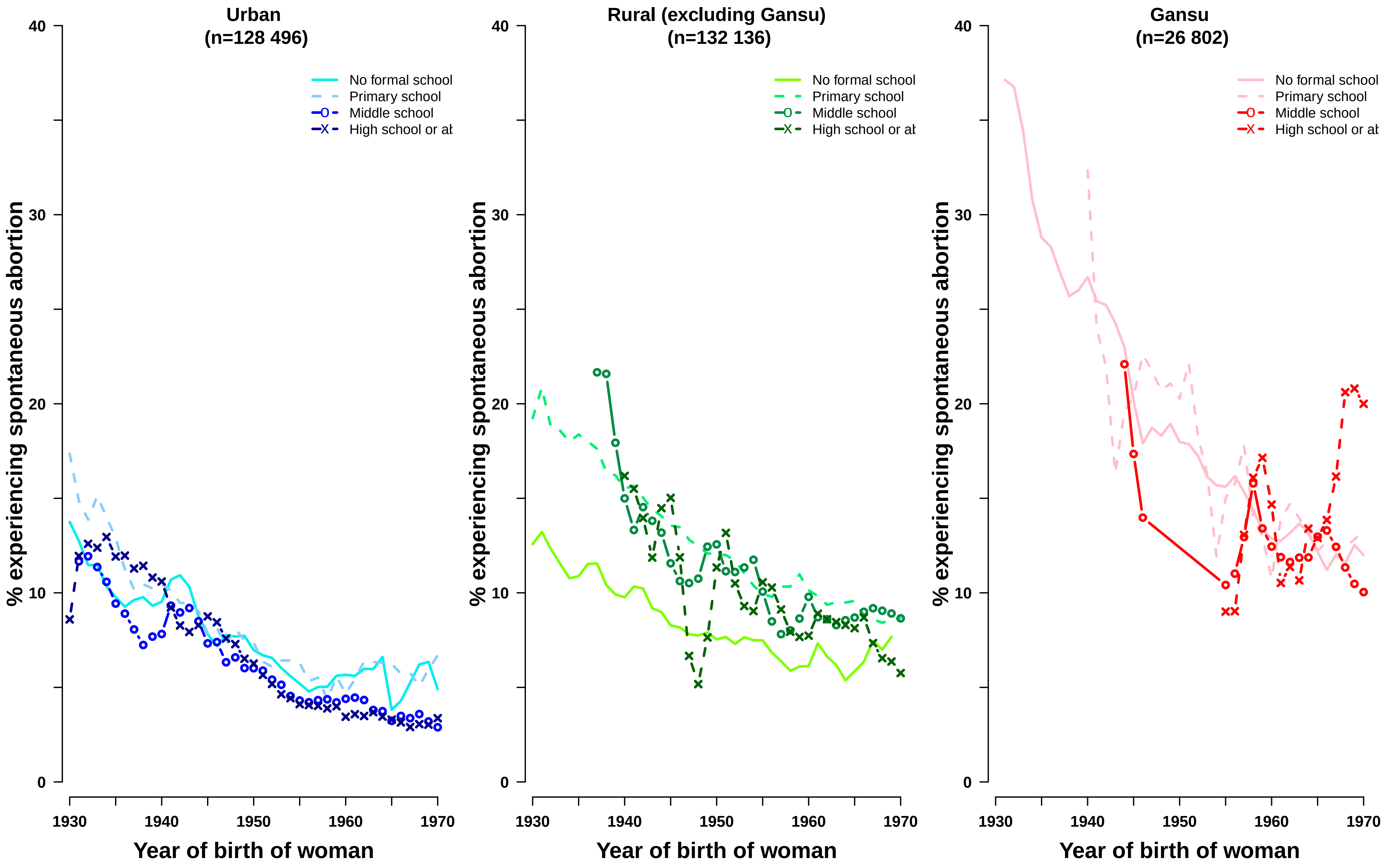

**Supplementary table 1.** Demographic and reproductive characteristics, by area

| | Area | | | | | | | | | |
|---|---|---|---|---|---|---|---|---|---|---|
| | Urban (North → South) | | | | | Rural (Coastal → Inland) | | | | |
| **Characteristics** | **Harbin** | **Qingdao** | **Suzhou** | **Liuzhou** | **Haikou** | **Zhejiang** | **Hunan** | **Henan** | **Sichuan** | **Gansu** |
| Number of women | 34164 | 19854 | 30783 | 30640 | 18732 | 33583 | 33220 | 35192 | 34101 | 30317 |
| Birth cohort, mean (SD) | 1953 (11.1) | 1955 (10.3) | 1954 (10.5) | 1953 (10.1) | 1954 (11.6) | 1954 (9.6) | 1955 (10.2) | 1956 (10.0) | 1955 (10.3) | 1958 (10.5) |
| Age at interview (years), mean (SD) | 52.6 (11.1) | 51.0 (10.3) | 51.3 (10.4) | 53.2 (10.1) | 52.0 (11.6) | 51.7 (9.7) | 50.4 (10.3) | 49.8 (10.0) | 50.2 (10.3) | 47.7 (10.4) |
| Highest education, % | 52 | 31 | 7 | 38 | 30 | 2 | 7 | 11 | 6 | 4 |
| Age at menarche, mean (SD) | 15.0 (1.8) | 15.8 (2.0) | 15.6 (1.9) | 14.8 (2.0) | 15.7 (2.1) | 15.5 (1.7) | 15.6 (1.9) | 15.5 (2.0) | 15.7 (2.1) | 15.4 (1.9) |
| Number with at least one pregnancy | 33456 | 19739 | 30610 | 30212 | 18439 | 33379 | 32999 | 34950 | 33860 | 30145 |
| Number of live birth, mean (SD) | 1.7 (1.1) | 1.7 (1.0) | 1.8 (1.1) | 2.0 (1.3) | 2.4 (1.3) | 2.2 (1.1) | 2.6 (1.2) | 2.7 (1.3) | 2.0 (1.4) | 3.2 (1.5) |
| History of induced abortion, % | 70 | 58 | 62 | 77 | 68 | 53 | 33 | 45 | 62 | 3 |
| History of spontaneous abortion, % | 5.8 | 3.9 | 5.8 | 7.4 | 8.1 | 5.2 | 15.5 | 11.5 | 8.4 | 16.0 |
| No history of abortion, % | 73 | 61 | 65 | 80 | 72 | 56 | 44 | 53 | 67 | 19 |
| Number with at least one live birth | 33085 | 19691 | 30564 | 29938 | 18335 | 33345 | 32848 | 34902 | 33806 | 30105 |
| Age at first birth, mean (SD) | 25.3 (3.3) | 25.7 (2.4) | 22.9 (2.6) | 25.1 (3.5) | 25.1 (3.6) | 21.8 (2.7) | 21.8 (2.6) | 23.1 (2.4) | 22.8 (2.6) | 22.0 (2.6) |
| Average months of breast feeding, mean (SD) | 13.1 (8.1) | 11.7 (4.8) | 13.5 (7.3) | 11.0 (4.5) | 10.7 (4.0) | 11.8 (4.9) | 12.4 (4.4) | 21.5 (8.4) | 15.9 (7.7) | 20.8 (7.2) |
| Number of postmenopausal women | 10972 | 5485 | 8558 | 9981 | 6194 | 10075 | 8799 | 8387 | 9144 | 6196 |
| Age at menopause mean (SD) | 49.2 (4.0) | 49.7 (3.9) | 48.7 (4.4) | 49.0 (4.2) | 48.9 (4.3) | 49.1 (4.0) | 48.4 (4.5) | 48.4 (4.4) | 48.2 (4.6) | 47.9 (4.6) |
